# Hematopoietic Stem Cell Development, Niches, and Signaling Pathways

**DOI:** 10.1155/2012/270425

**Published:** 2012-07-30

**Authors:** Kamonnaree Chotinantakul, Wilairat Leeanansaksiri

**Affiliations:** ^1^Stem Cell Therapy and Transplantation Research Group, Suranaree University of Technology, Nakhon Ratchasima 30000, Thailand; ^2^School of Microbiology, Institute of Science, Suranaree University of Technology, Nakhon Ratchasima 30000, Thailand

## Abstract

Hematopoietic stem cells (HSCs) play a key role in hematopoietic system that functions mainly in homeostasis and immune response. HSCs transplantation has been applied for the treatment of several diseases. However, HSCs persist in the small quantity within the body, mostly in the quiescent state. Understanding the basic knowledge of HSCs is useful for stem cell biology research and therapeutic medicine development. Thus, this paper emphasizes on HSC origin, source, development, the niche, and signaling pathways which support HSC maintenance and balance between self-renewal and proliferation which will be useful for the advancement of HSC expansion and transplantation in the future.

## 1. Introduction

Hematopoietic stem cells (HSC) are adult stem cells that contain the potentiality in self-renew and differentiation into specialized blood cells that function in some biological activities: control homeostasis balance, immune function, and response to microorganisms and inflammation. HSCs can also differentiate into other specialized cell or so called plasticity such as adipocytes [1], cardiomyocytes [2], endothelial cells [3], fibroblasts/myofibroblasts [4], liver cells [5, 6], osteochondrocytes [7, 8], and pancreatic cells [9]. Most HSCs are in quiescent state within the niches that maintain HSC pool and will respond to the signals after the balance of blood cells or HSC pool is disturbed from either intrinsic or extrinsic stimuli.

In addition, HSCs have been studied extensively, especially, for the therapeutic purposes in the treatment of blood diseases, inherited blood disorders, and autoimmune diseases. Nonetheless, advanced development in this field needs knowledge in the biological studies as a background in performing strategy and maintaining of HSCs. Thus, HSC source, origin, niches for HSC pool, and signaling pathways, essential for the regulation of HSCs, will be discussed in this review.

## 2. HSCs Origin and Development

In the hematopoietic system, the discovery of HSCs has shed the light on stem cell biology studies including connection to other adult stem cells through the basic concepts of differentiation, multipotentiality, and self-renewal. In the early period of those discoveries, lethally irradiated animals were found to be rescued by spleen cells or marrow cells [17, 18]. After mouse bone marrow cells were transplanted into irradiated mice, the clonogenic mixed colony of hematopoietic cells (often composed of granulocyte/megakaryocyte and erythroid precursors) were formed within the spleen, which these colonies were then termed colony-forming unit spleen (CFU-S) [19]. Some colonies of primary CFU-S could reconstitute hematopoietic system in the secondary irradiated mice after receiving transplantation [20]. Initially, CFU-S was first proposed that it may be differentiated from HSC, but subsequently, CFU-S was demonstrated to be originated from more committed progenitor cells [21]. The discovery by Till and McCulloch embarked on a new journey toward many investigations to clarify HSC biology, functional characterization, purify, cultivation, and other stem cells research.

**Figure 1:**
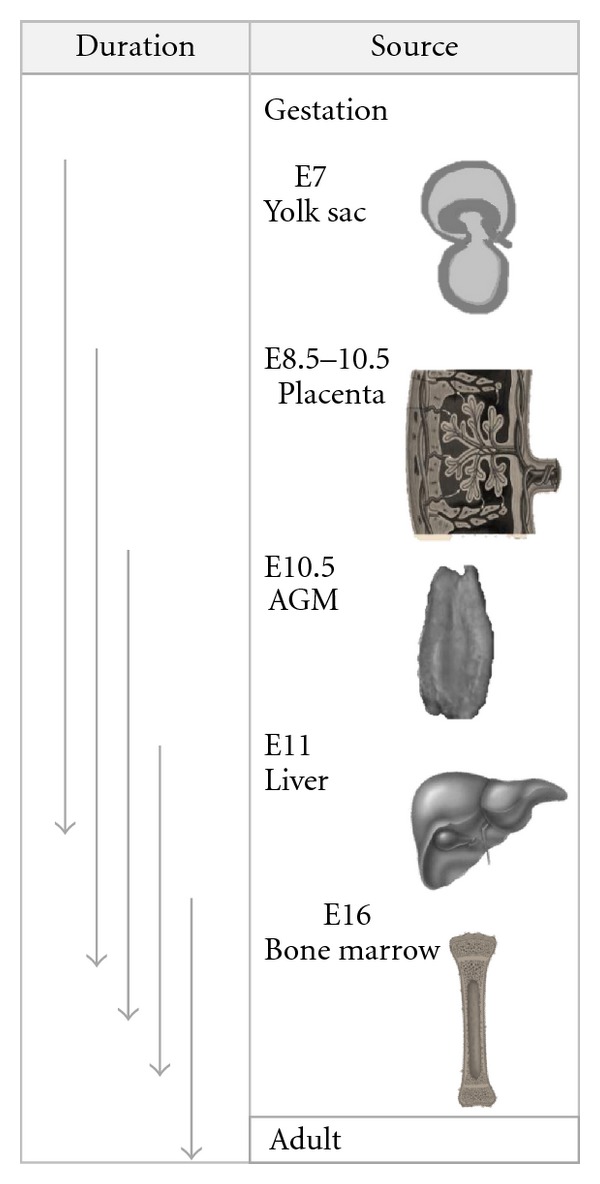
Source of blood cells during gestation through after birth. Intraembryonic yolk sac is the first site of blood cells observation at around E7.0–E7.5. The *de novo* hematopoiesis in the placenta and AGM occurs at nearly similar wave of gestation (around E8.5–E10.5) beforeit circulates into fetal liver where there is the large HSC pool during gestation. At around E16.5, the HSCs migrate and reside within the bone marrow which finally becomes the source of HSC in adult life (adapted from [10]).

Hematopoiesis and HSC development are the key role to improve efficient HSC expansion for the transplantations. Embryogenesis study has been performed to identify HSC origin and activity from various anatomical sites of several kinds of animals such as zebrafish, chicken, and mouse including human embryos model have been emerging. Initially, Moore and Metcalf showed that hematopoietic cells in the yolk sac could generate hematopoietic progenitors that restricted to only erythroid and myeloid lineages [22]. Moreover, the Runx1 (transcription factor for the onset of definitive hematopoiesis) was first identified to express at embryonic day 7.5 (E7.5) in the yolk sac, the chorionic mesoderm, and parts of allantoic mesoderm [23]. However, HSCs found in the yolk sac lacked the definitive hematopoietic stem cells which did not show long-term hematopoietic reconstitution activity in mouse embryo prior to E11.5 [24]. On the other hand, long-term repopulating HSCs (LT-HSCs) were shown to increase largely in the aorta-gonad mesonephros (AGMs) region of the mouse embryo including the serially transplantable irradiated mice, suggesting that AGM region is the first site for HSCs detection [24, 25]. Vitelline and umbilical arteries were also endowed with hematopoietic potential [26]. The presence of HSC phenotype in the embryo was supported by the evidence that a high number of nonerythroid progenitors with high-proliferative potential was observed from which the liver rudiment has been removed [27]. A dense population of CD34^+^ cells adhering to the ventral side of the aortic endothelium within the embryonic compartment was shown to display a cell-surface and molecular phenotype of primitive hematopoietic progenitors (CD45^+^, CD34^+^, CD31^+^, CD38^−^, negative for lineage markers, GATA-2^+^, GATA-3^+^, c-myb^+^, SCL/TAL1^+^, c-kit^+^, flk-1/KDR^+^) [28, 29]. Moreover, the autonomously emergence of myelolymphoid lineage from progenitors was found in splanchnopleural mesoderm and derived aorta within the human embryo proper, while restricted progenitors were generated in the yolk sac [30]. Altogether, AGM region in the embryo is suggested as the source of definitive hematopoiesis as the generation occurs between E10.5 and E12.0 with the enhance activity of HSC after mid-day 11 of gestation [10, 31–33]. Even though, the main source of fetal hematopoiesis was considered in AGM including vitelline and umbilical arteries, the question is raised whether the rare population produced in those regions would be enough for the distribution into fetal liver for alternative development of enormous HSCs before the transition of hematopoiesis continues to occur in the fetal thymus and bone marrow in postnatal life. Recently, the placenta, an extraembryonic organ, has been considered as the other hematopoietic organ for *de novo* hematopoiesis [34, 35]. This may be due to the physiology of the placenta containing highly vascularized blood vessels, and cytokines and growth factors rich environment for proper microenvironment of hematopoiesis and development [36]. Additionally, privilege site within the placenta may hide the HSCs from the promoting signal into differentiation stage. However, there is no experimental evidence to support that HSCs are generated *de novo* in the extraembryonic tissues. Therefore, future works will be needed to elucidate this enigma. Summarization of the source of blood cells during gestation through adult life has been elucidated in Figure 1.

**Figure 2:**
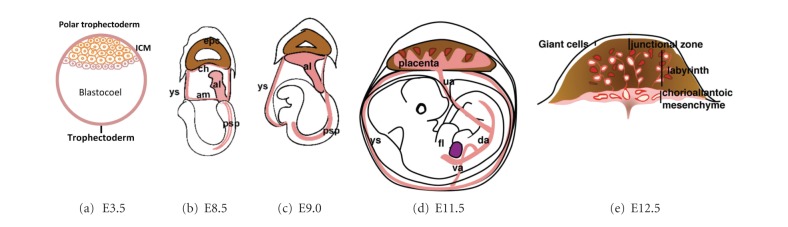
Mouse placenta development. (a) At E3.5 of early embryogenesis, blastocyst is formed, containing inner cell mass located at one side of the blastocoelic cavity and outer layer (trophectodermal epithelium) which give rise to the placenta. (b) Between E7.5–E8.25 mesodermal precursors originating from the primitive streak grow into the allantois (light grey) which then develops toward the ectoplacental cone (dark grey). (c) Chorioallantoic fusion between the allantoic and chorionic mesoderm at E8.5. After that, Chorionic villi and vasculature are formed producing and generates extensive villous branching called labyrinth. (d) At E11.5, umbilical cord is fully formed to connect the placenta with fetus where fetomaternal bloods circulate. (e) Cross-section of the placenta at E12.5 showing the chorioallantoic mesenchyme lies cover the placenta labyrinth with fetal vessels lined by fetal endothelium (dark vessels with lumen) and trophoblast lined by maternal blood spaces (grey vessels surrounded by dark trophoblasts). al, allantois; ch, chorion; am, amnion; epc, ectoplacental cone; ys, yolk sac; psp, para-aortic splanchnopleura; da, dorsal aorta; ua, umbilical artery; va, vitelline artery; fl, fetal liver (modified from [11]).

The origin of HSC in the placenta is being questioned. Understanding how theplacenta develops might be useful to define the source and the niches supporting HSC development. Mouse and human placentas are anatomically similar and its genes have analogous identity [37, 38]. The placenta is formed from trophectoderm, mesodermal tissues, chorionic mesoderm, and allantois (Figure 2) [39]. At E8.5 of mouse gestation, the allantois develops and fuses with chorionic mesoderm through its distal part generating the chorioallantoic mesenchyme in the chorionic plate and continuing to form the fetal vascular compartment of the placental labyrinth, while the proximal part becomes the umbilical cord [11].

The umbilical cord (a constitution of the fetal arteries and veins that inserted within chorionic plate of the placenta) is attached to the center of fetal surface for uteroplacenta circulation through maternal blood. Maternal blood passes through the placenta from uterine arteries to spiral arteries in the maternal decidua. Thereafter, the maternal blood percolates through the villous tree in humans (or the labyrinth in mice) known as chorionic villi which created and lined by fetal trophoblast cells [37, 39]. The inner core of the chorionic villi consists of allantoic mesenchyme and vasculature which is continuous with that of the umbilical cord. The chorioallantoic vasculature connects the placenta via the dorsal aorta and fetal liver through the umbilical cord vessels. These regions are localized by an equally dense network of fetal capillaries where the fetomaternal exchange occurs [37].

Because of the mesoderm layer gives rise to all blood cells, the chorionic and allantoic mesoderms are considered as the origin of HSC in the placenta. This can be explained by the observation that hematopoietic potential emerging from both tissues and has been identified with myeloerythroid potential [40]. In addition, hematopoietic cells (CD34^+^CD45^+^) collected from placental villi stroma and highly expression of CD45^+^ cells that appear to be budding from the vasculature have been found from human placenta during midgestation [35]. Moreover, cells harvested from term human placenta vessels and tissues could generate human hematopoietic repopulation of nonobese diabetic (NOD)-SCID mice, which harbored and/or amplified in vascular labyrinth placenta niche [35]. These observations imply that the placenta is the HSC source along with umbilical cord blood. At E10.5, first HSC emerge in the dorsal aorta before the onset of heart beat where the circulation has not been formed. One study showed that in the absence of heat beat in Ncx1 (the sodium and calcium exchange pump1) knockout embryos, the HSC development was verified to initiate in the placental vasculature [41]. Additionally, multilineage hematopoietic potential could be obtained from placentas of Ncx1 knockout embryos. Thus, within the extraembryonic tissues, fetal HSCs were observed in placenta, vitelline, and umbilical arteries.

The true origin of HSC in the intraembryonic hematopoiesis remains controversial. One of the main hypotheses is hemangioblasts or hemogenic endothelial while the alternative model is mesodermal precursors. The blood islands originated in the yolk sac are derived from mesodermal cell aggregates, which contain the ability to differentiate into both hematopoietic and endothelial cells. The common precursor by those lineages is suggested to be so called the hemangioblast [42]. Hematopoietic phenotype originated from hemogenic endothelium has been found in avian and mouse during ontogeny [43, 44]. Imaging and cell-tracking study explored that hemogenic endothelial cells could give rise to hematopoietic cells [45]. By time-lapse imaging study in single-cell mouse mesodermal cells demonstrated that it could generate endothelial sheet colonies and some colonies developed the hematopoietic morphology that upregulating the blood-specific proteins CD45, CD41, and CD11b and losing their intact morphology. Recently, this evidence has been supported by the observation on time-lapse confocal imaging from live mouse aorta showing that HSCs (Sca^+^, c-kit^+^, CD41^+^) could emerge directly from ventral aortic endothelial cells [46]. Moreover, Oberlin and colleagues proved that the origin of adult bone marrow HSCs which most of them derived were from the vascular endothelial-cadherin ancestor [47]. Taken together, these studies pinpoint the evidence that definitive hematopoietic stem and progenitor cells emerge from the hemogenic endothelium at the AGM region.

## 3. HSC Niches

Homing of HSC from other definitive hematopoiesis to fetal bone marrow is thought to involve some signaling factors such as stromal derived factor-1 (SDF-1 or CXCL12)/chemokine C-X-C receptor 4 (CXCR4) axis [48, 49]. Soluble factors are not only mediated in fetal bone marrow but also in adult bone marrow to maintain HSC in undifferentiated state and regulate HSC in proliferative and differentiated states within the specific microenvironments termed “niche” throughout the life [12]. Stem cell niche was first proposed by Schofield [21], with the later identification in *Drosophila melanogester*'s ovary to confirm the existence of HSC niche [50]. Germline stem cells resided in the Drosophila ovary that is surrounded by differentiated somatic cells have been shown to be essential for maintaining stem cells survival and division [50]. Thus, HSC niche is the special local environments of HSCs that maintains and controls HSCs function by regulating survival, self-renewal ability, and cell fate decision. Such molecules have been identified to be associated with HSC homing to bone marrow, for example, SDF1-*α*, *β*1-integrins, metalloproteinases (MMP), and serine-threonine protein phosphatase (PP)2A [51, 52]. By using real-time imaging, it is possible to explore the localization of HSCs with their function [53]. HSCs lodge in the endosteal surface, osteoblasts, and blood vessels, particularly in trabecular regions, in the mouse calvaria. On the contrary, more mature cells reside away from the endosteum. Similarly, a study by developed *ex vivo* real-time imaging in irradiated mice show the homing and lodgment of transplantable HSCs in the endosteal region of the trabecular bone area where they respond to bone marrow damage by rapidly dividing [54].

**Figure 3:**
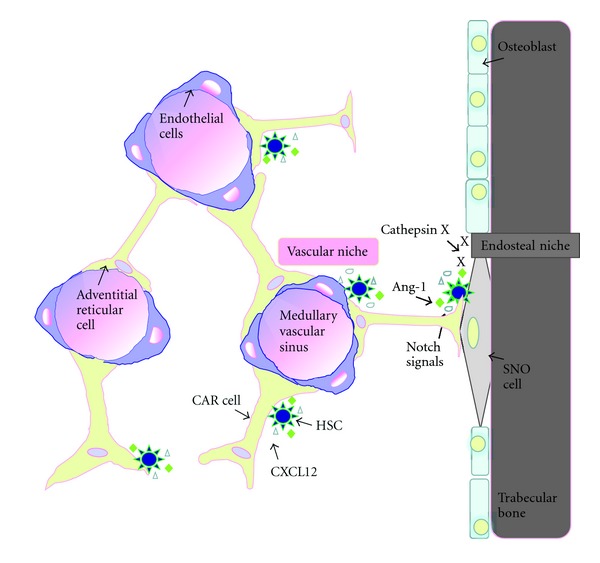
Candidate cellular niches mediated in maintenance and regulation of HSCs in bone marrow; endosteal and vascular niches. HSCs are in contact with SNO cells, bone-lining osteoblasts, within endosteal niche. Osteblasts produce several signal molecules such as Notch ligands, angiopoietin-1 (Ang-1), CXCL12, and cathepsin X mediated in control HSC pool and maintenance. Most HSCs are found in sinusoids, particularly adherence to CAR cells that surround sinusoidal endothelial cells (reticular niche). Similarly, CAR cells produce CXCL12 in association with CXCR4 signaling essentially for HSC maintenance (modified from [12]).

Recently HSCs niches are suggested to be mediated in two main microenvironments within bone marrow: endosteal niche and vascular niche (Figure 3). First, endosteal niche: osteoblasts derived from mesenchymal precursors are localized in the endosteal regions which are well vascularized. The activation of osteoblastic differentiation is in part mediated by HSC-derived bone morphogenic protein-2 (BMP-2) and BMP-6 [55]. Osteoblasts are suggested as the niche due to the finding that the number of osteoblasts is increased from parathyroid hormone activation and results in an increase HSCs number *in vivo* [56]. This signal was found to be activated through Jagged1, a serrate family of Notch ligand, on osteoblasts [57]. Study by Chitteti and colleagues supports this evidence and shows that enhancing hematopoiesis promoted by osteoblast via Notch signaling not only through Jagged1 upregulation, but also Notch2, Jagged2, Delta1 and 4, Hes1 and 5, and Deltex ligands [58]. Soluble factors produced from osteoblasts function in regulating HSC quiescence, HSC pool and fate such as angiopoietin-1 (Ang-1) [59], SDF-1 (CXCL12) [60], and osteopontin [61]. Recently, osteoblasts secreted cysteine protease cathepsin X have been found to catalyze the chemokine CXCL-12, a potent chemoattractive cytokine for HSCs, and ablate the attachment of CD34^+^ cells with the osteoblasts [62]. This result suggests the role of osteoblasts in regulate HSCs trafficking in the bone marrow.

A group of de Borros supports this hypothesis by showing that the 3D spheroid of noninduced and one week osteo-induce bone marrow stromal cell (active osteoblasts) formed an informative microenvironment that control migration, lodgment, and proliferation of HSCs [63]. Bone marrow endosteal cells, particularly, osteoblast-enriched ALCAM^+^Sca-1^−^ cells promoted LT-reconstitution activity of HSCs via the upregulation of genes related in homing and cell adhesion [64]. In addition, HSCs were found to adhere with spindle-shaped N-cadherin^+^ osteoblastic (SNO) cells which are a subpopulation of osteoblasts [65]. BMP receptor type IA mutant mice have been shown to increase in the number of SNO cells that correlated to an increase in HSC number [65]. Consistently, green fluorescent protein-positive (GFP^+^) HSCs derived from *Col2.3*-GFP^+^ transgenic mouse were found to attach to SNO cells but not all GFP^+^ HSCs were in contact with SNO cells showing that N-cadherin^−^ component might be the other niche for HSCs [54]. Cumulatively, osteoblasts and SNO cells are suggested as the niche for hematopoietic stem and progenitor cells where this microenvironment termed “Endosteal niche.”

Some observations have suggested that another niche, vascular niche, might involve in HSC maintenance within the bone marrow. Studies in osteoblast depletion demonstrated that there was a loss of B lymphopoiesis but not immediately loss of HSC number [66, 67] and few bone-marrow HSCs (CD150^+^CD48^−^CD41^−^lineage^−^) were localized to the endosteum [68]. Mice model defected in osteoblast function conferred no changes in LT-reconstitution function of HSCs [69]. Additionally, the loss of N-cadherin did not any effect on HSC maintenance and hematopoiesis [70]. Most HSCs in the bone marrow have been observed to reside in the sinusoid, where fenestrated endothelium persists and allows blood flow for an exchange of blood cells and small molecules. Taken together, the vascular niche is suggested as the other niche for HSC maintenance [68]. Bone-marrow endothelial cells have been proposed to play a role in HSC controlling within vascular niche. Primary CD31^+^ microvascular endothelial cells can restore hematopoiesis in mice when they receive bone-marrow lethal doses of irradiation [71]. Study by a group of Salter shows a consistent observation that endothelial progenitor cells injected in total body irradiated mice can stimulate HSC reconstitution and hematologic recovery [72]. Furthermore, selective activation of Akt in endothelial cells produced angiocrine factors mediated in the reconstitution, expansion, and maintenance of HSCs [73]. Nonetheless, constitutively activation of Akt, a binding ligand of phosphoinositide 3 in the phosphoinositide 3-kinase pathway, impaired engraftment ability and preferable generated leukemia in mice [74]. Sinusoidal endothelial cells are essential for engraftment of hematopoietic stem and progenitor cells (HSPCs) and restoration of hematopoiesis after myeloablation [75]. Angiocrine factors, such as Notch ligands, released by endothelial cells *in vivo* contributed to the replenishment of the LT-HSC pool and resulted in reconstitution of hematopoiesis [76]. Altogether, vascular niche containing endothelial cells is suggested as the major HSC pool and maintenance conferring proliferation and differentiation selection.

Additionally, Sugiyama and colleagues demonstrated that reticular cells located around the sinusoid endothelium could produce stromal cell-derived factor 1 (SDF-1, aka CXCL12) mediated in HSC niche [77]. These cells have been named CXCL12 abundant reticular cells (CAR cells). This study showed that almost all HSCs were found in contact with CAR cells and all HSCs allocated at endosteum were also found to be in contact with CAR cells, suggesting that these cells play a crucial role in HSC niches by homing HSCs in both vascular and endosteal niches [77]. CXCL12/CXCR4 signaling is essential in maintaining the HSC pool, development of B cells and plasmacytoid dendritic cells [78–81]. Moreover, short-term ablation of CAR cells resulted in the impairment of adipogenic and osteogenic differentiation. Thus, CAR cells are suggested as the adipo-osteogenic progenitors [82]. Study in CAR cell-depleted mice demonstrated that HSCs were reduced in number and cell size, which were more quiescent and highly expressed early myeloid selector genes [82]. CAR cells were suggested to coincide with CD146^+^ stromal progenitors that express CXCL12 and Ang-1. CD146^+^ cells could generate osteoblast that form bone and could function as skeletal progenitor cells [83]. Taken together, CAR cells provide or generate the hematopoietic microenvironment that link to the hematopoietic regulation in both vascular and endosteal niches.

## 4. Hierarchy of Human Hematopoiesis

**Figure 4:**
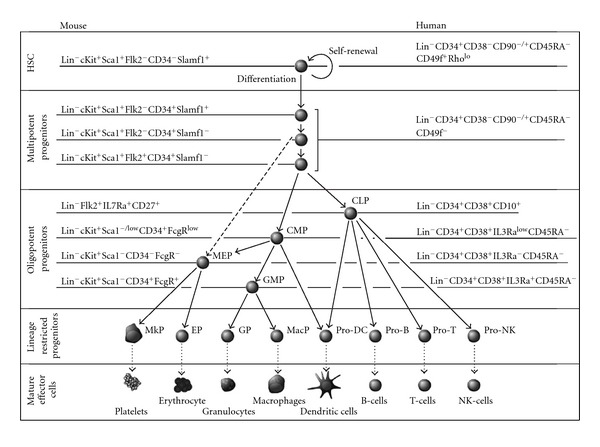
Hierarchy of hematopoiesis. The phenotypic cell surface marker of each population of mouse and human blood system is shown (modified from [13]). In the mouse hematopoiesis system, MPPs omit CMPs which directly give rise to MEPs unlink in the human system (dash line). CLP, common lymphoid progenitor; CMP, common myeloid progenitor; DC, dendritic cell; EP, erythrocyte progenitor; GMP, granulocyte/macrophage progenitor; GP, granulocyte progenitor; HSC, hematopoietic stem cell; MacP, macrophage progenitor; MEP, megakaryocyte/erythrocyte progenitor; MkP, megakaryocyte progenitor; NK, natural killer; Lin, lineage markers.

Based on the study of molecular marker expression by flow cytometry analysis has led the identification of each blood cell subpopulations in terms of their biology and potential when combined with other functional assays. As a result, schematic demonstration of hematopoietic hierarchy has been proposed (Figure 4) [13]. The origin of all blood cell in hematopoietic system is believed to be derived from HSCs that contain self-renewal capacity and give rise to multipotent progenitors (MPPs) which lose self-renewal potential but remain fully differentiate into all multilineages. MPPs further give rise to oligopotent progenitors which are common lymphoid and myeloid progenitors (CLPs and CMPs, resp.). All these oligopotent progenitors differentiate into their restricted lineage commitment: (1) CMPs advance to megakaryocyte/erythrocyte progenitors (MEPs), granulocyte/macrophage progenitors (GMPs), and dendritic cell (DC) progenitors, (2) CLPs give rise to T cell progenitors, B cell progenitors, NK cell progenitors and DC progenitors. Notably, DC progenitors (CD8*α* ^+^ DC, CD8*α* ^−^ DC, and plasmacytoid DC) could be derived from both CMPs and CLPs [84–86].

Among the isolation and characterization of HSCs and progenitors, CD34 molecule is the first widely chosen for the study by several researchers. CD34 is comprised in the CD34 family of cell-surface transmembrane proteins together with podocalyxin and endoglycan [87–89]. CD34 expression on blood cells is about 0.1–4.9% in human cord blood, bone marrow, and peripheral blood [90–92]. The first candidate human HSCs was a population of cells expressing CD34^+^CD90^+^(Thy-1)Lin^−^ which could give rise to T and B lymphocytes and myeloerythroid activities in both *in vitro* and *in vivo* human fetal thymus transplanted into SCID mice while some subset of CD34^−^, CD90^−^, Lin^−^ lacked of multipotent progenitors [93]. Further isolation of HSCs was based on the expression of CD38 [94, 95] and CD45RA [96]. This data could be concluded that Lin^−^CD34^+^CD38^−^CD90^+^CD45RA^−^ population enriches for human HSCs and the candidate human MPP fraction of multipotency with an incomplete self-renewal capacity is enriched in Lin^−^CD34^+^CD38^−^CD90^−^CD45RA^−^ population [97]. However, recently observation using HSC xenograft assay in NOD-SCID-IL2Rgc^−/−^ (NSG) mice has shown that both Lin^−^CD34^+^CD38^−^CD90^−^ CD45RA^−^ and Lin^−^CD34^+^CD38^−^CD90^+^CD45RA^−^ contain LT repopulating activity in secondary recipients with different frequency [98]. In addition, CD49f (integrin *α*6) marker has been shown as a specific HSC marker within Lin^−^CD34^+^CD38^−^CD45RA^−^ population which as single-sorted HSC is highly efficient in generating long-term multilineage grafts while the loss of CD49f expression results in the absence of long-term grafts [98]. Furthermore, Rhodamine-123 marker (efflux of the mitochondrial dye) is added to enrich for HSCs where high Rho efflux (Rho^lo^)Lin^−^CD34^+^CD38^−^CD90^+^CD45RA^−^ can also repopulate all blood lineages in secondary recipients [98]. Taken together, these results demonstrate that human HSCs are enriched in the Lin^−^CD34^+^CD38^−^CD90^+/−^CD45RA^−^Rho^lo^ population of hematopoietic cells (Figure 4).

## 5. Signaling Pathways in Self-Renewal**** and Maintenance of HSCs

The balance that controls between self-renewal and differentiation (or cell fate decision) of HSCs in the bone marrow is mediated by several factors. There are a number of animal models promoting the concept that the niches inside bone marrow provide the maintenance and regulation of HSCs by some microenvironmental-dependent signals. Most HSCs are in quiescent state (i.e., in G0/G1 phase of the cell cycle) [99], however, when the hematopoietic cells disturbance occurs, hematopoiesis system will respond by shutting down or turning on the regulators mediated in the regulations. Several pathways have been studied in relation to that circumstance which are SDF-1 (CXCL12)/CXCR4 signaling, BMP signaling, Mpl/Thrombopoietin (TPO) signaling, Tie2/Ang-1 signaling, hedgehog and Notch signaling, as well as Wingless (Wnt) signaling.

### 5.1. SDF-1 (CXCL12 )/CXCR4 Signaling Pathway

Stromal cell-derived factor 1 (SDF-1) is constitutively expressed in several organs including lung, liver, skin, and bone marrow [100]. SDF-1 belongs to *α*-chemokines that functions as chemoattractant for both committed and primitive hematopoietic progenitors and regulates embryonic development including organ homeostasis [100]. There are two main splicing forms that have been identified, SDF-1*α* and SDF-1*β*, which ubiquitously expressed with highest levels detected in liver, pancreas, and spleen [101]. Additionally, another variant form, SDF-*γ*, has been characterized in the nervous system [102]. Subsequently, SDF-1*δ*, SDF-1*ε*, and SDF-*ϕ* have been identified with highly expression in pancreases and lower levels detection in heart, kidney, liver, and spleen [103]. SDF-1 counteracts with its cognate receptor, CXCR4 that expresses widely in numerous tissues including hematopoietic and endothelial cells to stimulate the physiological processes. SDF-1/CXCR4 signaling plays a critical role during embryonic development by regulating B-cell lymphopoiesis, myelopoiesis in bone marrow and heart ventricular septum formation [104–106]. In addition, SDF-1 has been shown to be mediated in the recruitment of endothelial progenitor cells (EPCs) from the bone marrow through a CXCR4 dependent mechanism suggesting the functional role in vasculogenesis in which EPCs could form blood vessels [107]. A number of observations demonstrated that there was an increase in SDF-1 expression the ischemic sites [108, 109]. More evidence demonstrated that locally injection of SDF-1 augmented vasculogenesis and subsequently contributed to ischemic neovascularization *in vivo* by promoting EPC recruitment in ischemic tissues [110]. Recently, Liu and colleagues have shown that signal of SDF-1/CXCR4 together with CXCR7 can increase the mobilization and paracrine actions of mesenchymal stem cells (MSCs) ischemic kidneys under hypoxia condition [111]. Moreover, SDF-1/CXCR4 not only plays a role in HSC maintenance but also regulates HSC attachment within the niche. The mechanism mediated in this regulation was found to be activated through matrix metalloproteinase-9 that mediated in the releasing of soluble Kit-ligand [112]. Inactivation or deletion of CXCR4 in mice resulted in HSC pool reduction and hyperproliferation responsive to HSC defections [77, 78]. Tzeng and colleagues also confirmed the role of SDF-1 in HSC maintenance by demonstrating that a conditional SDF-1-deficient mice conferred an impairment in HSC quiescence and endosteal niche localization [113].

### 5.2. BMP Signaling Pathway

Bone morphogenic proteins (BMPs) are a group of growth factors that belongs to a TGF-*β* family member [114]. BMPs are mainly produced by osteoclasts in HSC niche [115]. During embryogenesis, BMP-4 regulates hematopoietic lineage commitment from mesodermal cell, while HSC number and function within bone marrow niche is controlled by Bmp-4 during adult life [116, 117]. Knowledge of BMP signaling and receptor related adult HSC within bone marrow has been studied in a small number and is elusive. BMP signaling impairment displayed an increase in the niche size, leading to the enhancement in the number of HSCs [65]. Differential response of HSC to soluble BMPs observed by a group of Bhatia showed that higher concentrations of BMP-2, BMP-4 and BMP-7 maintained human CB HSCs *in vitro* while at lower concentrations of BMP-4-induced proliferation and differentiation of HSCs [118].

### 5.3. c-Mpl/TPO Signaling Pathway

**Figure 5:**
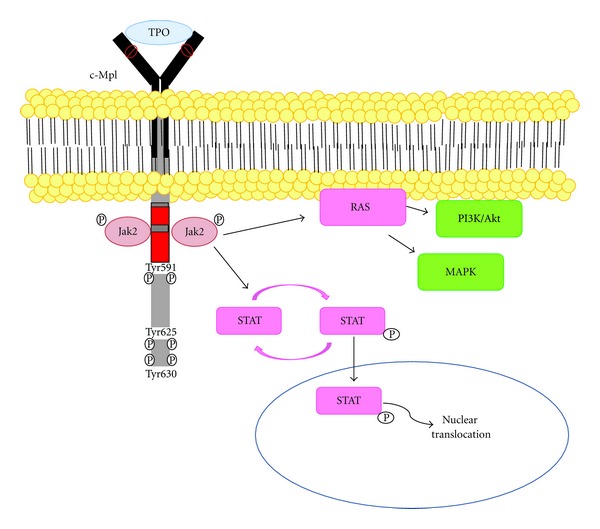
c-Mpl/TPO signaling pathway. TPO signals to its receptor, c-Mpl, and induces the downstream signaling cascades: STATs, PI3K, MAPKs, and extracellular signal regulated kinases-1 and -2 (modified from [14, 15]).

c-Mpl and its ligand, thrombopoietin (TPO), are known to regulate megakaryopoiesis [119]. c-Mpl receptor is expressed mainly on HSCs, with a lesser extent on megakaryocytic progenitors, megakaryocytes and platelets [120]. Various tissues expressing c-Mpl are mediated in hematopoiesis, including bone marrow, spleen, and fetal liver [14]. Based on the crystallographic EPO receptor study and its analogy to the TPO receptor have led to the postulation that TPO initiates the signal transduction by binding to the c-Mpl at the distal part, which in turn a homodimer of c-Mpl becomes active [15]. Consequently, Janus kinase 2 (JAK2) can phosphorylate tyrosine residues within the receptor itself which at least two tyrosine residues, Tyr625 and Tyr630, are phosphorylated on c-Mpl [15, 122], thereby stimulating the downstream cascade STATs, PI3K, the mitogen-activated protein kinases (MAPKs), and extracellular signal regulated kinases-1 and -2 (Figure 5) [123, 124]. c-Mpl/TPO signaling involved in postnatal steady-state HSC maintenance and cell-cycle progression at the endosteal surface [125, 126]. Mpl-expressed LT-HSCs were found in correlation to cell cycle quiescence and that was closely associated with TPO-producing osteoblastic cells in the bone marrow [125]. Additionally, the inhibitory adaptor protein Lnk was suggested as a negative regulator of JAK2 in HSCs following TPO stimulation, in which HSC quiescence and self-renewal controls were predominantly through Mpl [127]. Therefore, TPO/Mpl/JAK2/Lnk pathway can be concluded as a gatekeeper for HSC quiescence. Recently, TPO knock-in RAG2^−/−^ *γ*c^−/−^ mice has been shown to improve human engraftment in the bone marrow and maintenance of HSPCs pool by serial transplantation [128]. Taken together, TPO has an important function in maintenance and self-renewal of HSCs.

### 5.4. Tie2/Ang-1 Signaling

Angiopoietin-1 (Ang-1) is the ligand of Tie2, a receptor tyrosine kinase, which expresses predominantly on osteoblastic cells in endosteum [59] and in MSCs [83]. Interaction of Tie2 with its ligand, Ang-1, resulted in tightly adhesion of HSCs to the niche and become more quiescence [59]. Moreover, Ang-1 conferred the maintenance of LT-HSCs while Ang-2 did not antagonize the effects of Ang-1 on gene expression, Akt (aka protein B) phosphorylation [129].

### 5.5. Hedgehog Signaling Pathway

Hedgehog (Hh) is proposed as a negative regulator of the HSC quiescence [130]. Hh ligand binds to the transmembrane receptor Patched (Ptc) and subsequently allows the signaling function of a second transmembrane protein, Smoothened (Smo), essentially for the Hh signal to be active. Trowbridge and colleagues demonstrated that constitutive activation of the Hh signaling pathway in Ptc heterozygous (Ptc-1^+/−^) mice resulted in induction of cell cycling and expansion of primitive bone marrow hematopoietic cells [130]. To support this hypothesis, deletion of *Smo* in the in utero of transgenic mice was performed and the result demonstrated that there was an impaired stem cell self-renewal and the inhibition in engraftment activity of HSCs [131]. Furthermore, the common downstream positive effector of Hg signaling, Gli1, has been shown to play a critical role in normal and stress hematopoiesis [132]. Nonetheless, the discrepancies on Hg role in hematopoiesis were shown in some studies claiming that the conditional loss of *Smo* within adult HSCs is dispensable for hematopoiesis [133, 134]. These conflicts might be due to the difference of the mice model and conditional system used to impair Hg signaling.

### 5.6. Notch Signaling Pathway

Notch signaling plays a key role in several fundamental functions including proliferation, differentiation and cell fate decision [135, 136]. Four notch receptors (Notch 1–4) and five ligands (Jagged1-2 and Delta-like 1, 3, and 4) have been identified in mammals [137]. Cells expressing Notch ligands or engineered immobilized Notch ligands could maintain or enhance HSC self-renewal in the culture [138, 139]. Some investigations demonstrated that there were an impaired HSCs differentiation both *in vitro* [140, 141] and *in vivo* [139, 141] studies following interaction of Notch receptors and Notch ligands. Transcription factor act upstream of the Notch signaling cascade, Hes2, was shown to be essential in HSCs formation in zebrafish embryos when *hes2* expression was knockdown, whereas HSC formation could be rescued by the activation of Notch signal [142]. One study showed that an increase in *in vitro* maintenance of hematopoietic functions and repopulating potential on osteoblasts and Lineage^−^Sca-1^+^CD117^+^ (LSK) cells coculture was mediated by the up-regulation of Notch signal (Notch2, Jagged1 and 2, Delta1 and 4, Hes1 and 5, and Deltex) [58]. Taken together, these studies support the role of Notch signaling mediated in HSC hematopoiesis and maintenance. In the contrary, some investigations proposed that Notch signaling was not important for HSC self-renewal and maintenance [143, 144]. Inactivation of Notch1 and Jagged1 in bone marrow progenitors and bone marrow stroma, respectively, did not impair HSC maintenance and reconstitution [144]. The inhibition of Notch1–4 signaling via a developed dominant-negative Mastermind-like1 construct was transfected into LSK and demonstrated similar result of LT reconstitution in bone marrow compared to LSK control, except for T-cells [143]. Nevertheless, the study by Kim and colleague explored the important of Notch in normal hematopoiesis [145]. Mind bomb (Mib)-1, which regulates the endocytosis of Notch ligands and activation, was inactivated in mice leading to myeloproliferative disease (MPD). Surprisingly, when transplanted with wild-type bone marrow cells into the Mib1-null microenvironment, it results in a *de novo* MPD. The MPD progression was suppressed by transplantable Notch activating cells, suggesting that MPD develops from the nonhematopoietic microenvironmental cells with defective Notch signaling. Therefore, Notch signaling is indeed required for normal hematopoiesis. Santaguida and colleague developed *JunB*-defcient mice which resulted in impairment of Notch and transforming growth factor-*β* (TGF-*β*) signaling, in part via the transcriptional regulation of Hes1 [146]. This study showed an increase in LT-HSCs proliferation and differentiation without impairing their self-renewal *in vivo*, suggesting that LT-HSC proliferation rate is not exclusively compelling to self-renewal activity and maintenance of HSC in the BM niches.

### 5.7. Wnt Signaling Pathway

**Figure 6:**
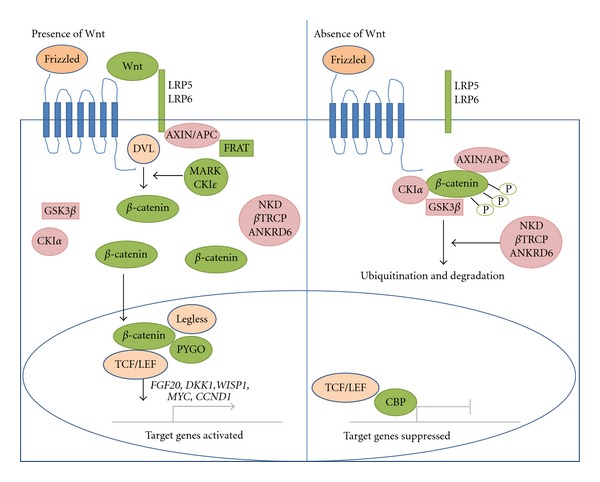
A canonical Wnt signaling pathway. In the presence of Wnt, the signals are transduced through Frizzled family receptors and LRP5/LRP6 coreceptor to the *β*-catenin signaling cascade which then stabilize hypophosphorylated *β*-catenin and interact with TCF/LEF, Legless and PYGO for target gene activations. MARK and CKI*ε* are the positive regulators of canonical Wnt pathway, while APC, AXIN1, AXIN2, CKI*α*, NKD1, NKD2, *β*TRCP1, *β*TRCP2, and ANKRD6 are negative regulators. In the absence of Wnt, *β*-catenin-forming complex with AXIN and APC is phosphorylates, leading to be a target for ubiquitination and degradation by proteasome (adapted from [16]).

Notch signaling is involved in the cross-talk with other pathways particularly Wnt signaling not only in hematopoiesis [147] but also in other cellular development [136, 148–151]. In addition, Wnt signaling pathway is mediated in the regulation of stem cell fate and maintenance of mouse ESCs and human ESCs in undifferentiated state [152, 153]. There are at least two independent pathway comprised in Wnt signaling: canonical Wnt and noncanonical Wnt signaling pathways. The canonical Wnt signal interacts with Frizzled (Fz) receptors and single-pass co-receptors LDL-receptor-related proteins 5 and 6 (LRP 5 and 6). The Fz protein contains a conserved motif, a cysteine rich domain (CRD) located on the extracellular domain that binds to multiple Wnts with a high affinity (Figure 6) [154]. Specifically, Dishevelled (DVL) is phosphorylated by casein kinase I*ε* (CKI*ε*), which then binds typically to FRAT and confers the assembly between Fz to DVL (Fz-DVL complex) and LRP5/6 to AXIN and FRAT (LRP5/6-AXIN-FRAT complex) [155, 156]. After that, *β*-catenin is stabilized and released from phosphorylation by CKI*α* and GSK3*β*. Then, *β*-catenin forms the complex with T-cell factor/lymphoid enhancer binding factor (TCF/LEF) family transcription factors and also with Legless family docking proteins (BCL9 and BCL9L) associated with PYGO family coactivators for stabilization and nuclear accumulation [157–159]. Typically, the downstream effectors for transcriptional activation target genes are *FGF20*, *DKK1*, *WISP1*, *MYC*, and *CCND1 *[160–163]. In the absence of Wnt, *β*-catenin is destabilized by phosphorylation of CKI*α* and glycogen synthase kinase 3*β* (GSK 3*β*), which then resulted in a formation of a destruction complex facilitating by Axin (*β*-catenin-APC-AXIN) that is polyubiquitinated by *β*TRCP1 or *β*TRCP2 complex for the degradation by proteasome [16, 159].

In the second pathway, “noncanonical Wnt signal” exerts the independent *β*-catenin signaling. The Wnt subfamily members, for example, Wnt5a binds to the Frizzled receptor, stimulates downstream intracellular signaling, resulting in an increase in intracellular Ca2^+^, and then activates protein kinase C and calmodulin-dependent kinase [159]. The cross-talk between Notch and Wnt signaling pathways was found in the stabilizing *β*-catenin on bone-marrow stroma cells that promoted maintaining and self-renewal of HSCs [164]. Moreover, the induction of Jagged1 and delta-like 1 was observed in Wnt/*β*-catenin-activated bone-marrow stroma or in bone-marrow stroma cultured with Wnt3a-conditioned medium [164]. Mice lacking Wnt3a resulted in prenatal death [165]. Moreover, Wnt3a deficiency reduced the number of HSCs in fetal liver and impaired the repopulating activity *in vivo* [165]. However, the exact role of Wnt signaling pathway in regulation of HSCs remains a controversy. Some studies demonstrated that constitutive activation of Wnt/*β*-catenin in transgenic mice resulting in the multilineage differentiation block and loss of repopulating stem cell activity due to the induction of quiescent stem cells entering into cell cycle and arresting their differentiation [166, 167]. In contrast to previous works, the administration of an inhibitor of GSK-3*β* *in vivo* displayed the enhancement in the recovery of hematopoietic cells for neutrophil and megakaryocytic lineages as well as primitive LSK cell population together with the upregulation of *Wnt*, *Notch,* and *Hedgehog* genes [168].

Furthermore, inhibition of Wnt signaling in HSCs by overexpression of the paninhibitor of canonical Wnt signaling, Dickkopf1 (Dkk1), resulted in the induction of cell cycling and reduction in repopulating ability in transplanted induction mice [169]. When the inhibitor of GSK-3*β*, 6-bromoindirubin 3′-oxime was used to treat CB-CD34^+^ cells, cell cycle progression was delayed including promoted engraftment of *ex vivo*-expanded HSCs [170]. Cumulatively, these studies suggest the positive regulatory role of Wnt/*β*-catenin signal on the proliferative or repopulating activity of HSCs. 12/15-lipoxygenase-mediated unsaturated fatty acid metabolism has been implicated in canonical Wnt-related signaling in the maintenance of LT-HSC quiescence and number [171]. Taken together, the canonical Wnt signal is mediated in the regulation of HSC function by maintaining quiescence and balance in proliferation.

## 6. Concluding Remarks

HSCs have been studied extensively for HSC source, hematopoiesis, biological functions, and signaling pathways related to the maintenance and regulation of HSCs. Advance researches such as imaging system clearly provide useful information on tracking the HSC origin, pool, and transplantation outcome in the mouse models. The observation of molecular mechanisms downstream the signaling cascade of self-renewing and proliferation of HSCs will also provide the knowledge through the new discovery in the treatment of diseases including the development in the performing a large scale preparation of HSCs for clinical transplantation. In addition, the signaling pathways will also provide understanding insight into the cancer stem cells which are now challenging scientists to explore their possible strategy for the treatments.

## References

[1] Sera Y, LaRue AC, Moussa O (2009). Hematopoietic stem cell origin of adipocytes. *Experimental Hematology*.

[2] Pozzobon M, Bollini S, Iop L (2010). Human bone marrow-derived CD133^+^ cells delivered to a collagen patch on cryoinjured rat heart promote angiogenesis and arteriogenesis. *Cell Transplantation*.

[3] Elkhafif N, El Baz H, Hammam O (2011). CD133^+^ human umbilical cord blood stem cells enhance angiogenesis in experimental chronic hepatic fibrosis. *Acta Pathologica, Microbiologica et Immunologica*.

[4] Ebihara Y, Masuya M, LaRue AC (2006). Hematopoietic origins of fibroblasts: II. In vitro studies of fibroblasts, CFU-F, and fibrocytes. *Experimental Hematology*.

[5] Khurana S, Mukhopadhyay A (2008). In vitro transdifferentiation of adult hematopoietic stem cells: an alternative source of engraftable hepatocytes. *Journal of Hepatology*.

[6] Sellamuthu S, Manikandan R, Thiagarajan R (2011). In vitro trans-differentiation of human umbilical cord derived hematopoietic stem cells into hepatocyte like cells using combination of growth factors for cell based therapy. *Cytotechnology*.

[7] Dominici M, Pritchard C, Garlits JE, Hofmann TJ, Persons DA, Horwitz EM (2004). Hematopoietic cells and osteoblasts are derived from a common marrow progenitor after bone marrow transplantation. *Proceedings of the National Academy of Sciences of the United States of America*.

[8] Mehrotra M, Rosol M, Ogawa M, LaRue AC (2010). Amelioration of a mouse model of osteogenesis imperfecta with hematopoietic stem cell transplantation: microcomputed tomography studies. *Experimental Hematology*.

[9] Minamiguchi H, Ishikawa F, Fleming PA (2008). Transplanted human cord blood cells generate amylase-producing pancreatic acinar cells in engrafted mice. *Pancreas*.

[10] Coskun S, Hirschi KK (2010). Establishment and regulation of the HSC niche: roles of osteoblastic and vascular compartments. *Birth Defects Research C*.

[11] Gekas C, Rhodes KE, van Handel B, Chhabra A, Ueno M, Mikkola HKA (2010). Hematopoietic stem cell development in the placenta. *International Journal of Developmental Biology*.

[12] Nagasawa T, Omatsu Y, Sugiyama T (2011). Control of hematopoietic stem cells by the bone marrow stromal niche: the role of reticular cells. *Trends in Immunology*.

[13] Seita J, Weissman IL (2010). Hematopoietic stem cell: self-renewal versus differentiation. *Wiley Interdisciplinary Reviews*.

[14] Chou FS, Mulloy JC (2011). The thrombopoietin/MPL pathway in hematopoiesis and leukemogenesis. *Journal of Cellular Biochemistry*.

[15] Geddis AE (2010). Megakaryopoiesis. *Seminars in Hematology*.

[16] Katoh M, Katoh M (2007). WNT signaling pathway and stem cell signaling network. *Clinical Cancer Research*.

[17] Jacobson LO, Simmons EL, Marks EK, Eldredge JH (1951). Recovery from radiation injury. *Science*.

[18] Lorenz E, Uphoff D, REID TR, Shelton E (1951). Modification of irradiation injury in mice and guinea pigs by bone marrow injections. *Journal of the National Cancer Institute*.

[19] Till JE, Mc EA (1961). A direct measurement of the radiation sensitivity of normal mouse bone marrow cells. *Radiation Research*.

[20] Siminovitch L, Mcculloch EA, Till JE (1963). The distribution of colony-forming cells among spleen colonies. *Journal of Cellular Physiology*.

[21] Schofield R (1978). The relationship between the spleen colony-forming cell and the haemopoietic stem cell. A hypothesis. *Blood Cells*.

[22] Moore MA, Metcalf D (1970). Ontogeny of the haemopoietic system: yolk sac origin of in vivo and in vitro colony forming cells in the developing mouse embryo. *British Journal of Haematology*.

[23] Zeigler BM, Sugiyama D, Chen M, Guo Y, Downs KM, Speck NA (2006). The allantois and chorion, when isolated before circulation or chorio-allantoic fusion, have hematopoietic potential. *Development*.

[24] Medvinsky A, Dzierzak E (1996). Definitive hematopoiesis is autonomously initiated by the AGM region. *Cell*.

[25] Müller AM, Medvinsky A, Strouboulis J, Grosveld F, Dzierzak E (1994). Development of hematopoietic stem cell activity in the mouse embryo. *Immunity*.

[26] de Bruijn MFTR, Speck NA, Peeters MCE, Dzierzak E (2000). Definitive hematopoietic stem cells first develop within the major arterial regions of the mouse embryo. *The EMBO Journal*.

[27] Huyhn A, Dommergues M, Izac B (1995). Characterization of hematopoietic progenitors from human yolk sacs and embryos. *Blood*.

[28] Labastie MC, Cortés F, Roméo PH, Dulac C, Péault B (1998). Molecular identity of hematopoietic precursor cells emerging in the human embryo. *Blood*.

[29] Tavian M, Coulombel L, Luton D, Clemente HS, Dieterlen-Lièvre F, Péault B (1996). Aorta-associated CD34^+^ hematopoietic cells in the early human embryo. *Blood*.

[30] Tavian M, Robin C, Coulombel L, Péault B (2001). The human embryo, but not its yolk sac, generates lympho-myeloid stem cells: mapping multipotent hematopoietic cell fate in intraembryonic mesoderm. *Immunity*.

[31] Chen MJ, Yokomizo T, Zeigler BM, Dzierzak E, Speck NA (2009). Runx1 is required for the endothelial to haematopoietic cell transition but not thereafter. *Nature*.

[32] de Bruijn MFTR, Ma X, Robin C, Ottersbach K, Sanchez MJ, Dzierzak E (2002). Hematopoietic stem cells localize to the endothelial cell layer in the midgestation mouse aorta. *Immunity*.

[33] Taylor E, Taoudi S, Medvinsky A (2010). Hematopoietic stem cell activity in the aorta-gonad-mesonephros region enhances after mid-day 11 of mouse development. *International Journal of Developmental Biology*.

[34] Bárcena A, Kapidzic M, Muench MO (2009). The human placenta is a hematopoietic organ during the embryonic and fetal periods of development. *Developmental Biology*.

[35] Robin C, Bollerot K, Mendes S (2009). Human placenta is a potent hematopoietic niche containing hematopoietic stem and progenitor cells throughout development. *Cell Stem Cell*.

[36] Cross JC (2005). How to make a placenta: mechanisms of trophoblast cell differentiation in mice—a review. *Placenta*.

[37] Georgiades P, Fergyson-Smith AC, Burton GJ (2002). Comparative developmental anatomy of the murine and human definitive placentae. *Placenta*.

[38] Cox B, Kotlyar M, Evangelou AI (2009). Comparative systems biology of human and mouse as a tool to guide the modeling of human placental pathology. *Molecular Systems Biology*.

[39] Rossant J, Cross JC (2001). Placental development: lessons from mouse mutants. *Nature Reviews Genetics*.

[40] Corbel C, Salaün J, Belo-Diabangouaya P, Dieterlen-Lièvre F (2007). Hematopoietic potential of the pre-fusion allantois. *Developmental Biology*.

[41] Rhodes KE, Gekas C, Wang Y (2008). The emergence of hematopoietic stem cells isInitiated in the placental vasculature in the absence of circulation. *Cell Stem Cell*.

[42] Murray PDF (1932). The development in vitro of the blood of the early chick embryo. *Proceedings of the Royal Society*.

[43] Jaffredo T, Gautier R, Eichmann A, Dieterlen-Lièvre F (1998). Intraaortic hemopoietic cells are derived from endothelial cells during ontogeny. *Development*.

[44] Nishikawa SI, Nishikawa S, Kawamoto H (1998). In vitro generation of lymphohematopoietic cells from endothelial cells purified from murine embryos. *Immunity*.

[45] Eilken HM, Nishikawa SI, Schroeder T (2009). Continuous single-cell imaging of blood generation from haemogenic endothelium. *Nature*.

[46] Boisset JC, Van Cappellen W, Andrieu-Soler C, Galjart N, Dzierzak E, Robin C (2010). In vivo imaging of haematopoietic cells emerging from the mouse aortic endothelium. *Nature*.

[47] Oberlin E, Hafny BE, Petit-Cocault L, Souyri M (2010). Definitive human and mouse hematopoiesis originates from the embryonic endothelium: a new class of HSCs based on VE-cadherin expression. *International Journal of Developmental Biology*.

[48] Ara T, Tokoyoda K, Sugiyama T, Egawa T, Kawabata K, Nagasawa T (2003). Long-term hematopoietic stem cells require stromal cell-derived factor-1 for colonizing bone marrow during ontogeny. *Immunity*.

[49] Guo Y, Hangoc G, Bian H, Pelus LM, Broxmeyer HE (2005). SDF-1/CXCL12 enhances survival and chemotaxis of murine embryonic stem cells and production of primitive and definitive hematopoietic progenitor cells. *Stem Cells*.

[50] Xie T, Spradling AC (1998). decapentaplegic is essential for the maintenance and division of germline stem cells in the Drosophila ovary. *Cell*.

[51] Nilsson SK, Simmons PJ, Bertoncello I (2006). Hemopoietic stem cell engraftment. *Experimental Hematology*.

[52] Basu S, Ray NT, Atkinson SJ, Broxmeyer HE (2007). Protein phosphatase 2A plays an important role in stromal cell-derived factor-1/CXC chemokine ligand 12-mediated migration and adhesion of CD34^+^ cells. *Journal of Immunology*.

[53] Lo Celso C, Fleming HE, Wu JW (2009). Live-animal tracking of individual haematopoietic stem/progenitor cells in their niche. *Nature*.

[54] Xie Y, Yin T, Wiegraebe W (2009). Detection of functional haematopoietic stem cell niche using real-time imaging. *Nature*.

[55] Jung Y, Song J, Shiozawa Y (2008). Hematopoietic stem cells regulate mesenchymal stromal cell induction into osteoblasts thereby participating in the formation of the stem cell niche. *Stem Cells*.

[56] Calvi LM, Adams GB, Weibrecht KW (2003). Osteoblastic cells regulate the haematopoietic stem cell niche. *Nature*.

[57] Weber JM, Forsythe SR, Christianson CA (2006). Parathyroid hormone stimulates expression of the Notch ligand Jagged1 in osteoblastic cells. *Bone*.

[58] Chitteti BR, Cheng YH, Poteat B (2010). Impact of interactions of cellular components of the bone marrow microenvironment on hematopoietic stem and progenitor cell function. *Blood*.

[59] Arai F, Hirao A, Ohmura M (2004). Tie2/angiopoietin-1 signaling regulates hematopoietic stem cell quiescence in the bone marrow niche. *Cell*.

[60] Taichman RS (2005). Blood and bone: two tissues whose fates are intertwined to create the hematopoietic stem-cell niche. *Blood*.

[61] Stier S, Ko Y, Forkert R (2005). Osteopontin is a hematopoietic stem cell niche component that negatively regulates stem cell pool size. *Journal of Experimental Medicine*.

[62] Staudt ND, Aicher WK, Kalbacher H (2010). Cathepsin X is secreted by human osteoblasts, digests CXCL-12 and impairs adhesion of hematopoietic stem and progenitor cells to osteoblasts. *Haematologica*.

[63] de Barros APDN, Takiya CM, Garzoni LR (2010). Osteoblasts and bone marrow mesenchymal stromal cells control hematopoietic stem cell migration and proliferation in 3D in vitro model. *PLoS ONE*.

[64] Nakamura Y, Arai F, Iwasaki H (2010). Isolation and characterization of endosteal niche cell populations that regulate hematopoietic stem cells. *Blood*.

[65] Zhang J, Niu C, Ye L (2003). Identification of the haematopoietic stem cell niche and control of the niche size. *Nature*.

[66] Zhu J, Garrett R, Jung Y (2007). Osteoblasts support B-lymphocyte commitment and differentiation from hematopoietic stem cell. *Blood*.

[67] Visnjic D, Kalajzic Z, Rowe DW, Katavic V, Lorenzo J, Aguila HL (2004). Hematopoiesis is severely altered in mice with an induced osteoblast deficiency. *Blood*.

[68] Kiel MJ, Radice GL, Morrison SJ (2007). Lack of evidence that hematopoietic stem cells depend on N-cadherin-mediated adhesion to osteoblasts for their maintenance. *Cell Stem Cell*.

[69] Ma YD, Park C, Zhao H (2009). Defects in osteoblast function but no changes in long-term repopulating potential of hematopoietic stem cells in a mouse chronic inflammatory arthritis model. *Blood*.

[70] Kiel MJ, Acar M, Radice GL, Morrison SJ (2009). Hematopoietic stem cells do not depend on N-cadherin to regulate their maintenance. *Cell Stem Cell*.

[71] Li B, Bailey AS, Jiang S, Liu B, Goldman DC, Fleming WH (2010). Endothelial cells mediate the regeneration of hematopoietic stem cells. *Stem Cell Research*.

[72] Salter AB, Meadows SK, Muramoto GG (2009). Endothelial progenitor cell infusion induces hematopoietic stem cell reconstitution in vivo. *Blood*.

[73] Kobayashi H, Butler JM, O’Donnell R (2010). Angiocrine factors from Akt-activated endothelial cells balance self-renewal and differentiation of haematopoietic stem cells. *Nature Cell Biology*.

[74] Kharas MG, Okabe R, Ganis JJ (2010). Constitutively active AKT depletes hematopoietic stem cells and induces leukemia in mice. *Blood*.

[75] Hooper AT, Butler JM, Nolan DJ (2009). Engraftment and reconstitution of hematopoiesis is dependent on VEGFR2-mediated regeneration of sinusoidal endothelial cells. *Cell Stem Cell*.

[76] Butler JM, Nolan DJ, Vertes EL (2010). Endothelial cells are essential for the self-renewal and repopulation of notch-dependent hematopoietic stem cells. *Cell Stem Cell*.

[77] Sugiyama T, Kohara H, Noda M, Nagasawa T (2006). Maintenance of the hematopoietic stem cell pool by CXCL12-CXCR4 chemokine signaling in bone marrow stromal cell niches. *Immunity*.

[78] Nie Y, Han YC, Zou YR (2008). CXCR4 is required for the quiescence of primitive hematopoietic cells. *Journal of Experimental Medicine*.

[79] Noda M, Omatsu Y, Sugiyama T, Oishi S, Fujii N, Nagasawa T (2011). CXCL12-CXCR4 chemokine signaling is essential for NK-cell development in adult mice. *Blood*.

[80] Kohara H, Omatsu Y, Sugiyama T, Noda M, Fujii N, Nagasawa T (2007). Development of plasmacytoid dendritic cells in bone marrow stromal cell niches requires CXCL12-CXCR4 chemokine signaling. *Blood*.

[81] Nagasawa T (2006). Microenvironmental niches in the bone marrow required for B-cell development. *Nature Reviews Immunology*.

[82] Omatsu Y, Sugiyama T, Kohara H (2010). The essential functions of adipo-osteogenic progenitors as the hematopoietic stem and progenitor cell niche. *Immunity*.

[83] Sacchetti B, Funari A, Michienzi S (2007). Self-renewing osteoprogenitors in bone marrow sinusoids can organize a hematopoietic microenvironment. *Cell*.

[84] Traver D, Akashi K, Manz M (2000). Development of CD8*α*-positive dendritic cells from a common myeloid progenitor. *Science*.

[85] Manz MG, Traver D, Akashi K (2001). Dendritic cell development from common myeloid progenitors. *Annals of the New York Academy of Sciences*.

[86] Manz MG, Traver D, Miyamoto T, Weissman IL, Akashi K (2001). Dendritic cell potentials of early lymphoid and myeloid progenitors. *Blood*.

[87] Doyonnas R, Kershaw DB, Duhme C (2001). Anuria, omphalocele, and perinatal lethality in mice lacking the CD34-related protein podocalyxin. *Journal of Experimental Medicine*.

[88] Sassetti C, Van Zante A, Rosen SD (2000). Identification of endoglycan, a member of the CD34/podocalyxin family of sialomucins. *Journal of Biological Chemistry*.

[89] Sassetti C, Tangemann K, Singer MS, Kershaw DB, Rosen SD (1998). Identification of Podocalyxin-like protein as a high endothelial venule ligand for L-selectin: parallels to CD34. *Journal of Experimental Medicine*.

[90] Pranke P, Hendrikx J, Debnath G (2005). Immunophenotype of hematopoietic stem cells from placental/umbilical cord blood after culture. *Brazilian Journal of Medical and Biological Research*.

[91] Hossle JP, Seger RA, Steinhoff D (2002). Gene therapy of hematopoietic stem cells: strategies for improvement. *News in Physiological Sciences*.

[92] Krause DS, Fackler MJ, Civin CI, May WS (1996). CD34: structure, biology, and clinical utility. *Blood*.

[93] Baum CM, Weissman IL, Tsukamoto AS, Buckle AM, Peault B (1992). Isolation of a candidate human hematopoietic stem-cell population. *Proceedings of the National Academy of Sciences of the United States of America*.

[94] Bhatia M, Wang JCY, Kapp U, Bonnet D, Dick JE (1997). Purification of primitive human hematopoietic cells capable of repopulating immune-deficient mice. *Proceedings of the National Academy of Sciences of the United States of America*.

[95] Hao QL, Shah AJ, Thiemann FT, Smogorzewska EM, Crooks GM (1995). A functional comparison of CD34^+^CD38^−^ cells in cord blood and bone marrow. *Blood*.

[96] Mayani H, Dragowska W, Lansdorp PM (1993). Characterization of functionally distinct subpopulations of CD34^+^ cord blood cells in serum-free long-term cultures supplemented with hematopoietic cytokines. *Blood*.

[97] Majeti R, Park CY, Weissman IL (2007). Identification of a hierarchy of multipotent hematopoietic progenitors in human cord blood. *Cell Stem Cell*.

[98] Notta F, Doulatov S, Laurenti E, Poeppl A, Jurisica I, Dick JE (2011). Isolation of single human hematopoietic stem cells capable of long-term multilineage engraftment. *Science*.

[99] Fleming WH, Alpern EJ, Uchida N, Ikuta K, Spangrude GJ, Weissman IL (1993). Functional heterogeneity is associated with the cell cycle status of murine hematopoietic stem cells. *Journal of Cell Biology*.

[100] Ratajczak MZ, Zuba-Surma E, Kucia M, Reca R, Wojakowski W, Ratajczak J (2006). The pleiotropic effects of the SDF-1-CXCR4 axis in organogenesis, regeneration and tumorigenesis. *Leukemia*.

[101] Shirozu M, Nakano T, Inazawa J (1995). Structure and chromosomal localization of the human stromal cell-derived factor 1 (SDF1) gene. *Genomics*.

[102] Gleichmann M, Gillen C, Czardybon M (2000). Cloning and characterization of SDF-1*γ*, a novel SDF-1 chemokine transcript with developmentally regulated expression in the nervous system. *European Journal of Neuroscience*.

[103] Yu L, Cecil J, Peng SB (2006). Identification and expression of novel isoforms of human stromal cell-derived factor 1. *Gene*.

[104] Balabanian K, Lagane B, Infantino S (2005). The chemokine SDF-1/CXCL12 binds to and signals through the orphan receptor RDC1 in T lymphocytes. *Journal of Biological Chemistry*.

[105] Moepps B, Braun M, Knopfle K (2000). Characterization of a Xenopus laevis CXC chemokine receptor 4: implications for hematopoietic cell development in the vertebrate embryo. *European Journal of Immunology*.

[106] Braun M, Wunderlin M, Spieth K, Knöchel W, Gierschik P, Moepps B (2002). Xenopus laevis stromal cell-derived factor 1: conservation of structure and function during vertebrate development. *Journal of Immunology*.

[107] Zheng H, Fu G, Dai T, Huang H (2007). Migration of endothelial progenitor cells mediated by stromal cell-derived factor-1*α*/CXCR4 via PI3K/Akt/eNOS signal transduction pathway. *Journal of Cardiovascular Pharmacology*.

[108] Ceradini DJ, Kulkarni AR, Callaghan MJ (2004). Progenitor cell trafficking is regulated by hypoxic gradients through HIF-1 induction of SDF-1. *Nature Medicine*.

[109] Yang Y, Tang G, Yan J (2008). Cellular and molecular mechanism regulating blood flow recovery in acute versus gradual femoral artery occlusion are distinct in the mouse. *Journal of Vascular Surgery*.

[110] Yamaguchi JI, Kusano KF, Masuo O (2003). Stromal cell-derived factor-1 effects on ex vivo expanded endothelial progenitor cell recruitment for ischemic neovascularization. *Circulation*.

[111] Liu H, Liu S, Li Y (2012). The role of SDF-1-CXCR4/CXCR7 axis in the therapeutic effects of hypoxia-preconditioned mesenchymal stem cells for renal ischemia/reperfusion injury. *PLoS One*.

[112] Heissig B, Hattori K, Dias S (2002). Recruitment of stem and progenitor cells from the bone marrow niche requires MMP-9 mediated release of Kit-ligand. *Cell*.

[113] Tzeng YS, Li H, Kang YL, Chen WG, Cheng W, Lai DM (2011). Loss of Cxcl12/Sdf-1 in adult mice decreases the quiescent state of hematopoietic stem/progenitor cells and alters the pattern of hematopoietic regeneration after myelosuppression. *Blood*.

[114] Reddi AH, Reddi A (2009). Bone morphogenetic proteins (BMPs): from morphogens to metabologens. *Cytokine and Growth Factor Reviews*.

[115] Garimella R, Tague SE, Zhang J (2008). Expression and synthesis of bone morphogenetic proteins by osteoclasts: a possible path to anabolic bone remodeling. *Journal of Histochemistry and Cytochemistry*.

[116] Goldman DC, Bailey AS, Pfaffle DL, Al Masri A, Christian JL, Fleming WH (2009). BMP4 regulates the hematopoietic stem cell niche. *Blood*.

[117] Durand C, Robin C, Bollerot K, Baron MH, Ottersbach K, Dzierzak E (2007). Embryonic stromal clones reveal developmental regulators of definitive hematopoietic stem cells. *Proceedings of the National Academy of Sciences of the United States of America*.

[118] Bhatia M, Bonnet D, Wu D (1999). Bone morphogenetic proteins regulate the developmental program of human hematopoietic stem cells. *Journal of Experimental Medicine*.

[119] Broudy VC, Kaushansky K (1995). Thrombopoietin, the c-mpl ligand, is a major regulator of platelet production. *Journal of Leukocyte Biology*.

[120] Debili N, Wendling F, Cosman D (1995). The MpI receptor is expressed in the megakaryocytic lineage from late progenitors to platelets. *Blood*.

[121] Livnah O, Stura EA, Middleton SA, Johnson DL, Jolliffe LK, Wilson IA (1999). Crystallographic evidence for preformed dimers of erythropoietin receptor before ligand activation. *Science*.

[122] Drachman JG, Kaushansky K (1997). Dissecting the thrombopoietin receptor: functional elements of the Mpl cytoplasmic domain. *Proceedings of the National Academy of Sciences of the United States of America*.

[123] Witthuhn BA, Quelle FW, Silvennoinen O (1993). JAK2 associates with the erythropoietin receptor and is tyrosine phosphorylated and activated following stimulation with erythropoietin. *Cell*.

[124] Tortolani PJ, Johnston JA, Bacon CM (1995). Thrombopoietin induces tyrosine phosphorylation and activation of the Janus kinase, JAK2. *Blood*.

[125] Yoshihara H, Arai F, Hosokawa K (2007). Thrombopoietin/MPL signaling regulates hematopoietic stem cell quiescence and interaction with the osteoblastic niche. *Cell Stem Cell*.

[126] Qian H, Buza-Vidas N, Hyland CD (2007). Critical role of thrombopoietin in maintaining adult quiescent hematopoietic stem cells. *Cell Stem Cell*.

[127] Bersenev A, Wu C, Balcerek J, Tong W (2008). Lnk controls mouse hematopoietic stem cell self-renewal and quiescence through direct interactions with JAK2. *Journal of Clinical Investigation*.

[128] Rongvaux A, Willinger T, Takizawa H (2011). Human thrombopoietin knockin mice efficiently support human hematopoiesis in vivo. *Proceedings of the National Academy of Sciences of the United States of America*.

[129] Gomei Y, Nakamura Y, Yoshihara H (2010). Functional differences between two Tie2 ligands, angiopoietin-1 and -2, in regulation of adult bone marrow hematopoietic stem cells. *Experimental Hematology*.

[130] Trowbridge JJ, Scott MP, Bhatia M (2006). Hedgehog modulates cell cycle regulators in stem cells to control hematopoietic regeneration. *Proceedings of the National Academy of Sciences of the United States of America*.

[131] Zhao C, Chen A, Jamieson CH (2009). Hedgehog signalling is essential for maintenance of cancer stem cells in myeloid leukaemia. *Nature*.

[132] Merchant A, Joseph G, Wang Q, Brennan S, Matsui W (2010). Gli1 regulates the proliferation and differentiation of HSCs and myeloid progenitors. *Blood*.

[133] Hofmann I, Stover EH, Cullen DE (2009). Hedgehog signaling is dispensable for adult murine hematopoietic stem cell function and hematopoiesis. *Cell Stem Cell*.

[134] Gao J, Graves S, Koch U (2009). Hedgehog signaling is dispensable for adult hematopoietic stem cell function. *Cell Stem Cell*.

[135] Artavanis-Tsakonas S, Rand MD, Lake RJ (1999). Notch signaling: cell fate control and signal integration in development. *Science*.

[136] Lin GL, Hankenson KD (2011). Integration of BMP, Wnt, and Notch signaling pathways in osteoblast differentiation. *Journal of Cellular Biochemistry*.

[137] Ranganathan P, Weaver KL, Capobianco AJ (2011). Notch signalling in solid tumours: a little bit of everything but not all the time. *Nature Reviews Cancer*.

[138] Ohishi K, Varnum-Finney B, Bernstein ID (2002). Delta-1 enhances marrow and thymus repopulating ability of human CD34^+^CD38^−^ cord blood cells. *Journal of Clinical Investigation*.

[139] Varnum-Finney B, Halasz LM, Sun M, Gridley T, Radtke F, Bernstein ID (2011). Notch2 governs the rate of generation of mouse long- and short-term repopulating stem cells. *Journal of Clinical Investigation*.

[140] Varnum-Finney B, Brashem-Stein C, Bernstein ID (2003). Combined effects of Notch signaling and cytokines induce a multiple log increase in precursors with lymphoid and myeloid reconstituting ability. *Blood*.

[141] Stier S, Cheng T, Dombkowski D, Carlesso N, Scadden DT (2002). Notch1 activation increases hematopoietic stem cell self-renewal in vivo and favors lymphoid over myeloid lineage outcome. *Blood*.

[142] Rowlinson JM, Gering M (2010). Hey2 acts upstream of Notch in hematopoietic stem cell specification in zebrafish embryos. *Blood*.

[143] Maillard I, Koch U, Dumortier A (2008). Canonical notch signaling is dispensable for theMaintenance of adult hematopoietic stem cells. *Cell Stem Cell*.

[144] Mancini SJC, Mantei N, Dumortier A, Suter U, MacDonald HR, Radtke F (2005). Jagged1-dependent Notch signaling is dispensable for hematopoietic stem cell self-renewal and differentiation. *Blood*.

[145] Kim YW, Koo BK, Jeong HW (2008). Defective Notch activation in microenvironment leads to myeloproliferative disease. *Blood*.

[146] Santaguida M, Schepers K, King B (2009). JunB protects against myeloid malignancies by limiting hematopoietic stem cell proliferation and differentiation without affecting self-renewal. *Cancer Cell*.

[147] Clements WK, Kim AD, Ong KG, Moore JC, Lawson ND, Traver D (2011). A somitic Wnt16/Notch pathway specifies haematopoietic stem cells. *Nature*.

[148] Chalamalasetty RB, Dunty WC, Biris KK (2011). The Wnt3a/*β*-catenin target gene Mesogenin1 controls the segmentation clock by activating a Notch signalling program. *Nature Communications*.

[149] Han S, Dziedzic N, Gadue P, Keller GM, Gouon-Evans V (2011). An endothelial cell niche induces hepatic specification through dual repression of Wnt and notch signaling. *Stem Cells*.

[150] Peter IS, Davidson EH (2011). A gene regulatory network controlling the embryonic specification of endoderm. *Nature*.

[151] Kim DW, Lee JS, Yoon ES, Lee BI, Park SH, Dhong ES (2011). Influence of human adipose-derived stromal cells on Wnt signaling in organotypic skin culture. *Journal of Craniofacial Surgery*.

[152] Sato N, Meijer L, Skaltsounis L, Greengard P, Brivanlou AH (2004). Maintenance of pluripotency in human and mouse embryonic stem cells through activation of Wnt signaling by a pharmacological GSK-3-specific inhibitor. *Nature Medicine*.

[153] Woll PS, Morris JK, Painschab MS (2008). Wnt signaling promotes hematoendothelial cell development from human embryonic stem cells. *Blood*.

[154] Wu CH, Nusse R (2002). Ligand receptor interactions in the Wnt signaling pathway in Drosophila. *Journal of Biological Chemistry*.

[155] Wong HC, Bourdelas A, Krauss A (2003). Direct binding of the PDZ domain of Dishevelled to a conserved internal sequence in the C-terminal region of Frizzled. *Molecular Cell*.

[156] Tolwinski NS, Wehrli M, Rives A, Erdeniz N, DiNardo S, Wieschaus E (2003). Wg/Wnt signal can be transmitted through arrow/LRP5,6 and axin independently of Zw3/Gsk3*β* activity. *Developmental Cell*.

[157] Kramps T, Peter O, Brunner E (2002). Wnt/Wingless signaling requires BCL9/legless-mediated recruitment of pygopus to the nuclear *β*-catenin-TCF complex. *Cell*.

[158] Katoh M, Katoh M (2003). Identification and characterization of human BCL9L gene and mouse Bcl9l gene in silico. *International Journal of Molecular Medicine*.

[159] Miller JR, Hocking AM, Brown JD, Moon RT (1999). Mechanism and function of signal transduction by the Wnt/B-catenin and Wnt/Ca2^+^ pathways. *Oncogene*.

[160] Chamorro MN, Schwartz DR, Vonica A, Brivanlou AH, Cho KR, Varmus HE (2005). FGF-20 and DKK1 are transcriptional targets of *β*-catenin and FGF-20 is implicated in cancer and development. *The EMBO Journal*.

[161] Pennica D, Swanson TA, Welsh JW (1998). WISP genes are members of the connective tissue growth factor family that are up-regulated in Wnt-1-transformed cells and aberrantly expressed in human colon tumors. *Proceedings of the National Academy of Sciences of the United States of America*.

[162] He TC, Sparks AB, Rago C (1998). Identification of c-MYC as a target of the APC pathway. *Science*.

[163] Tetsu O, McCormick F (1999). *β*-catenin regulates expression of cyclin D1 in colon carcinoma cells. *Nature*.

[164] Kim JA, Kang YJ, Park G (2009). Identification of a stroma-mediated Wnt/*β*-catenin signal promoting self-renewal of hematopoietic stem cells in the stem cell niche. *Stem Cells*.

[165] Luis TC, Weerkamp F, Naber BAE (2009). Wnt3a deficiency irreversibly impairs hematopoietic stem cell self-renewal and leads to defects in progenitor cell differentiation. *Blood*.

[166] Scheller M, Huelsken J, Rosenbauer F (2006). Hematopoietic stem cell and multilineage defects generated by constitutive *β*-catenin activation. *Nature Immunology*.

[167] Kirstetter P, Anderson K, Porse BT, Jacobsen SEW, Nerlov C (2006). Activation of the canonical Wnt pathway leads to loss of hematopoietic stem cell repopulation and multilineage differentiation block. *Nature Immunology*.

[168] Trowbridge JJ, Xenocostas A, Moon RT, Bhatia M (2006). Glycogen synthase kinase-3 is an in vivo regulator of hematopoietic stem cell repopulation. *Nature Medicine*.

[169] Fleming HE, Janzen V, Lo Celso C (2008). Wnt signaling in the niche enforces hematopoietic stem cell quiescence and is necessary to preserve self-renewal in vivo. *Cell Stem Cell*.

[170] Ko K-H, Holmes T, Palladinetti P (2011). GSK-3*β* inhibition promotes engraftment of ex vivo-expanded hematopoietic stem cells and modulates gene expression. *Stem Cells*.

[171] Kinder M, Wei C, Shelat SG (2010). Hematopoietic stem cell function requires 12/15-lipoxygenase-dependent fatty acid metabolism. *Blood*.

